# Multicentric Retrospective Study on Safety and Efficacy of a Novel Injectable Poly‐l‐Lactic Acid for Buttocks Recontouring

**DOI:** 10.1111/jocd.16580

**Published:** 2024-10-24

**Authors:** Paola Rosalba Russo, Bruno Bovani, Francesca De Angelis, Riccardo Forte, Franco Vercesi, Giovanni Salti

**Affiliations:** ^1^ Private Practitioner, Esteemed Aesthetic Clinic Modena Italy; ^2^ Private Practitioner, Esculapio Perugia Italy; ^3^ Private Practitioner, DeA Center Laser & Plastic Surgery Clinic Naples Italy; ^4^ Private Practitioner, RF Clinics Como Italy; ^5^ Private Practitioner, Centro Medico Galeno Milan Italy; ^6^ Private Practitioner, Medlight Medical Institute Florence Italy

**Keywords:** aesthetic medicine, buttocks recontouring, efficacy and safety, off‐label use, poly‐l‐lactic acid

## Abstract

**Background:**

Poly‐l‐lactic acid (PLLA) injectables have gained increasing attention in aesthetic medicine due to their biocompatibility and long‐lasting effects. Although their primary application centers around facial rejuvenation, their potential for off‐label use in other body areas has been investigated, demonstrating promising outcomes in terms of both efficacy and safety.

**Aim:**

This study aims to assess the safety and efficacy of a novel 630 mg PLLA‐based filler (GANA X), in buttocks treatment.

**Methods:**

Six physicians treated 51 patients for buttock aesthetic treatment across six different medical facilities in Italy. A survey was filled by both physicians and patients regarding treatment safety, efficacy, and change in quality of life.

**Results:**

We reported minor or mild adverse events, self‐resolving within the next few days. Both physicians and patients confirmed notable aesthetic improvements following treatment, varying from moderate to significant enhancement. These effects endured throughout follow‐up visits spanning up to 24 months. Patient‐reported outcomes indicated elevated self‐esteem and improved quality of life posttreatment.

**Conclusions:**

The high level of satisfaction reported by both physicians and patients highlights the efficacy and tolerability of GANA X filler for buttocks treatment, encouraging their use and research for off‐label body areas.

## 1 Introduction

Concerns pertaining to physical appearance and rejuvenation are on the rise within our aging population, encompassing both women aiming to preserve their attractiveness and men desiring to retain robust physical traits [1]. The utilization of soft tissue fillers has gained substantial popularity as a minimally invasive cosmetic intervention, facilitating the augmentation of volume in regions affected by soft tissue loss attributed to age or disease, as well as the correction of wrinkles [2]. The prevalence of their application is consistently escalating, as evidenced by a surge in procedures from 1.6 million in 2011 to 3.4 million in 2020 in the United States [3]. Among different soft tissue fillers, poly‐l‐lactic acid (PLLA) has gathered increasing attention within the field of aesthetic surgery due to its biocompatibility and biodegradability properties, as well as its capacity to induce the generation of fresh collagen by eliciting a controlled tissue response [4]. Unlike other fillers that provide instant correction, as hyaluronic acid, PLLA fillers are more properly classified as biostimulators and result in a longer‐lasting volumetric augmentation, with effects observable over 2 years despite the full metabolization of PLLA microparticles after 9 months [5].

Poly‐l‐lactic acid (PLLA) initially received approval for soft tissue augmentation in Europe in 1999, primarily for addressing scars and wrinkles through cosmetic procedures. However, a retrospective evaluation reveals that the initial recommendations for its application, comprising aspects such as product reconstitution, hydration, injection sites, techniques, timing, and patient selection, were either insufficient or suboptimal [6]. By 2004, both European (EMA) and American (FDA) regulatory authorities extended the indications for PLLA usage to encompass substantial volume corrections for facial lipoatrophy (FLA), a type of lipodystrophy associated with the use of antiretroviral drugs for human immunodeficiency virus (HIV) [7]. This expansion in labeling coincided with the widespread adoption of significant modifications to the PLLA reconstitution and injection methods [8]. Four pivotal methodological factors underwent alteration to reduce side effects as nodules formation: resuspension volume was increased to more than 5 mL (with a common final volume of 8–9 mL), hydration time was prolonged to a range of 36–48 h, a dose of lidocaine was introduced into the preparation immediately before injection, and the injection site for PLLA shifted from the lower dermis to the uppermost layer of subcutaneous fat. Implementation of these protocol adjustments resulted in a reduction of complications [9, 10, 11]. By 2009, PLLA was FDA approved for facial volumization and wrinkles correction [12]. Since then, PLLA has been used extensively as a facial filler, showing long‐lasting results, high levels of patient satisfaction, and low percentage of mild adverse events [13, 14]. PLLA has been proved to be safe with few contraindications to the procedure, mainly relating to hypersensitivity to PLLA or other formulation components, the use of the product in patients with severe allergies, susceptibility to scarring or keloid formation, active inflammatory diseases, or with a chronic use of immunosuppressants and corticosteroids. It is also recommended to avoid the use in pregnant women and in people in anti‐coagulant therapy [15, 16].

Despite its initial approval for facial augmentation, PLLA underwent early investigations for applications beyond the face, such as the neck, chest, and hands, which remained connected to facial tightening and addressing photoaging concerns [17]. Presently, PLLA finds extensive utility in various body treatments aimed at augmenting volume, refining body contours, mitigating skin laxity, alleviating cellulite, addressing scars, and treating striae distensae across different body regions [1]. A multitude of studies have been dedicated to proving the effectiveness and safety of PLLA in nonfacial contexts. These investigations have encompassed diverse body areas, including the buttocks [18], arms [19], abdomen [20], knees [21], and thighs [22].

During the last 5 years women's attention increasingly focused on the gluteal region, defining feminine attractiveness especially on their “B” side. Gluteal augmentation requests dramatically increased for both surgical and nonsurgical procedures [23]. According to a recent survey, medical gluteal augmentation stands as the second most prevalent application of PLLA in the United States (42.4%), surpassed only by its use for treating HIV lipoatrophy (46.8%), emphasizing the importance of PLLA for off‐face treatments [24]. More recently, PLLA formulations have been marketed in larger vials, tailored to effectively address substantial depressed areas like the buttocks [1]. Other differences in new formulations rely on the size, porosity, and shape of PLLA microparticles that influence the degradation rate, impacting the overall success of the procedure [4]. Semi‐crystalline microspheres, for instance, exhibit a longer degradation rate than amorphous and porous PLLA fillers, resulting in a better result and a longer‐lasting effect [4]. Also PLLA composition impacts the degradation rate, with poly(d,l‐lactic acid) particles degrading faster than poly(l‐lactic acid) ones. Based on hydrolytic degradation kinetics at 37°C, amorphous and porous poly(d,l‐lactic acid) particles, as the ones found in AestheFill and Repart PLA, almost completely degraded, while for crystalline poly(l‐lactic acid) particles, as found in Sculptra and Gana V, the reduction in molecular weight was 62% and 78%, respectively [4].

In this study, we report our clinical experience with the use of GANA X, a novel 630 mg injectable PLLA filler for buttocks volumization and body contouring performed by six qualified aesthetic surgeons. The treatment safety and efficacy were assessed by qualified physicians and satisfactory metrics of patients and physicians were reported.

## 2 Materials and Methods

In total, 51 female patients (mean age 48.8 ± 8.9 years) were treated by six aesthetic surgeons treated across six different medical facilities in Italy between June 2019 and June 2022. The inclusion criteria for inclusion to the study were as follows: age between 18 and 60 years; request for improvement in gluteal shape, volume, or tone; no previous injectable materials in the gluteal area for at least 12 months; availability to attend at least two treatment sessions; commitment to a follow‐up period lasting 24 months; body mass index (BMI) of 25 or less. The exclusion criteria were weight variation of 3 kg or more during the study protocol and failure to follow up.

GANA X (PLLA powder, 630 mg/vial) from GANA R&D, Korea, was used in this study. The treatment was performed on upper gluteus, lateral depression (hip dip), cellulite dimples, contour irregularities, or skin tone and texture as summarized in Table 1. Vials were resuspended with 40–50 mL of sterile water and injected after only 1 h after reconstitution through fanning or retrograde linear techniques, by 18‐, 19‐, 20‐, or 22‐gauge needles, depending on the treated area (e.g., 18‐gauge for volumization, 22‐gauge for cellulite dimples). Injections were repeated with an interval of 6–12 weeks to enhance results and 8–12 months for maintenance, for 1–6 treatments. Treatment average duration was 30–60 min. The treatment was administered with a fanning technique, retro‐injecting PLLA in a vector fashion at one or two needle entry points per gluteus. The location of the injection was chosen based on the target area: upper‐inner quadrant for volumization, upper‐outer quadrant for contour and elevation, or the entire region of skin flaccidity for skin quality improvement [23]. The layer of injection was consistently subcutaneous across all treatments. For volumization purposes, the injections were administered in the medium‐deep subcutaneous fat tissue over the superficial lamina of the gluteal fascia. In contrast, for cellulite treatment, the injections were performed strictly in the subdermal layer, still within the subcutaneous tissue. It is important to avoid treating inflamed or infected areas, to ensure that injections are not administered into dermal vessels, to avoid overcorrection, and to refrain from performing the treatment in areas that have been previously treated with long‐term fillers. Not all patients received the same quantity of PLLA. The number of sessions ranged from one to five, and the amount of the active principle varied based on the intended goal and the anatomical area being treated.

**TABLE 1:** Treatment type, duration, and intervals performed by surgeons on the 51 patients in this study.

	(*n* pat/tot *n* pat)
Type of treatment	
Upper gluteus	90% (46/51)
Lateral depression (hip dip)	53% (27/51)
Cellulite dimples	39% (20/51)
Contour irregularities	41% (21/51)
Skin tone and texture	47% (24/51)
Number of treatments	
1	12% (1/49)
2	39% (19/49)
3	24% (12/49)
4	16% (8/49)
5	4% (2/49)
6	4% (2/49)
Treatment duration	
30 min	75% (38/51)
30–60 min	25% (13/51)
Interval between treatments	
6 weeks	16% (7/43)
8 weeks	63% (27/43)
12 weeks	7% (3/43)
3 months	2% (1/43)
4 months	9% (4/43)
6 months	2% (1/43)

Treatment assessment was performed for up to 24 months during follow‐up visits. Both patients and surgeons filled out a survey to assess adverse events (AE), efficacy of the treatment, and quality of life after treatment. All physicians filled the survey for the 51 treatments, whereas 49 patients filled the survey (two patients did not return the survey). Data were obtained through a signed consensus form.

The surveys evaluated the rate of AEs asking both physician and patients whether they experienced posttreatment AEs, and in which form (light, moderate, or extreme). Evaluated issues are presented in Table 2. Patients were asked about experienced pain during the treatment, and if they experienced any AEs during the following days, stating for how many days (1–2, 3–4, 5–7, 8–10, or over 10 days). To assess the efficacy of the treatment, the Global Aesthetic Improvement Scale (GAIS) was used [25] to evaluate the treatment results immediately after the treatment and during follow‐up visits in the following 24 months. Results were ranked by physicians and patients as “exceptionally improved,” “much improved,” “improved,” “no change,” or “worsened.” The survey evaluated the impact on patient's quality of life after the treatment by questions on self‐esteem, social life, sentiment about the treatment decision, and evaluation on treatment results. Questions were answered as “completely agree,” “moderately agree,” “moderately disagree,” or “completely disagree.” Another set of questions asked about the impact in the first days after treatment, as: regret, anxiety, sleepiness, fatigue, restriction of movements, usual activities, social context, or intimacy. Questions were answered as “never,” “rarely,” “sometimes,” or “often.”

**TABLE 2:** Survey posttreatment evaluation assessment for physicians and patients.

Physician posttreatment assessment	Patient posttreatment assessment
Acneiform eruptions	Pain during treatment
Visible nodules or palpable material	Swelling
Asymmetry	Soreness
Overcorrection	Bruising
Under correction	Inflammation
Needle mark	Tenderness
Inflammatory nodule	Pain
Tyndall effect	Numbness
Allergic reactions	Tingling
Granulomas	Pulsing sensation
Pain	Burning
Erythema‐edema	Lightheadedness
Ecchymosis‐hematoma	Headache
Bruising	Fever feeling
Vascular occlusion	
Covid‐19 vaccine‐related hypersensitivity reaction	

## 3 Results

After the GANA X treatment, both physicians and patients independently assessed the safety and efficacy of the treatment, and patients assessed the impact on quality of life.

**FIGURE 1:**
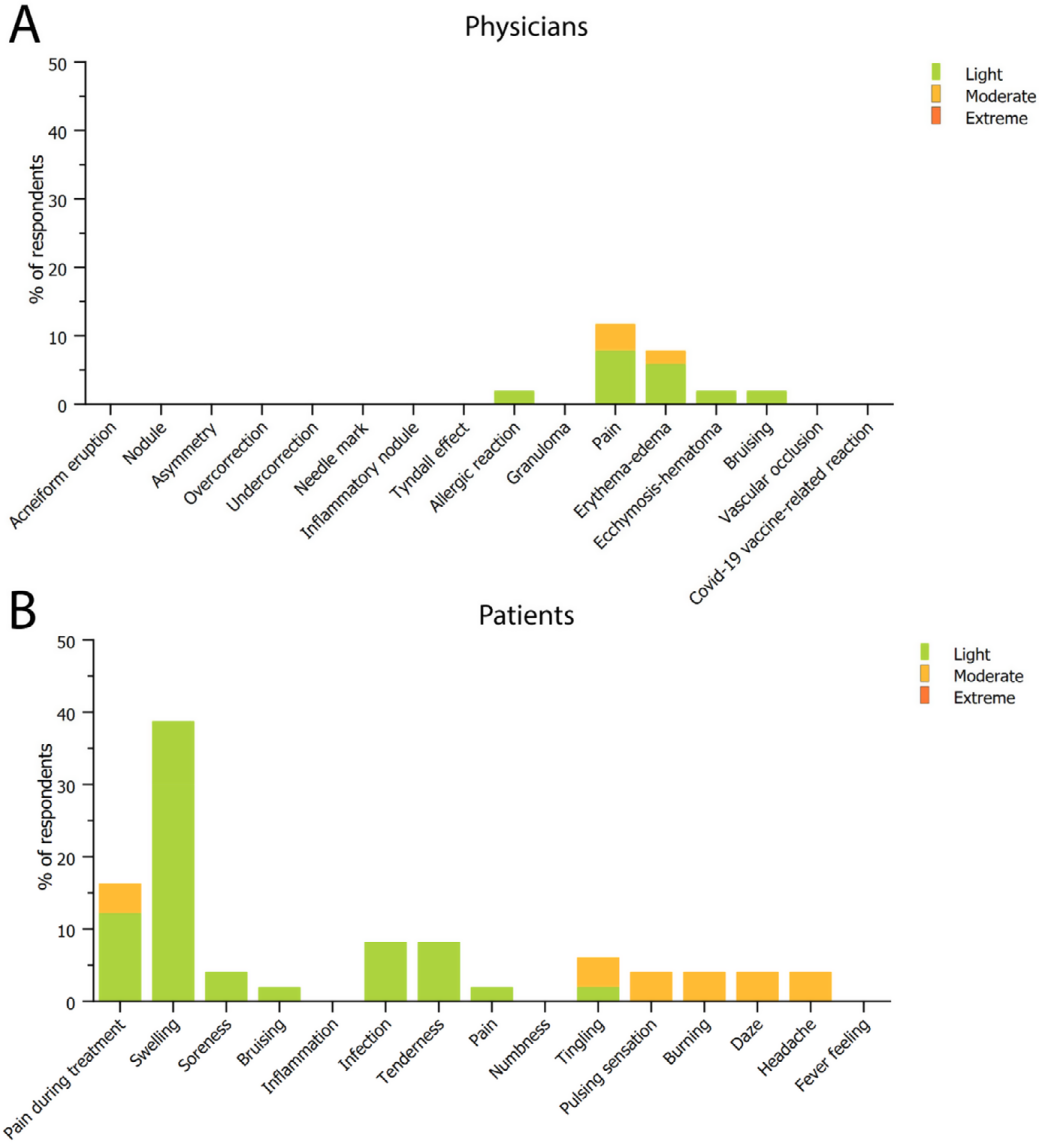
Adverse events (AEs) observed as reported by physicians (A), or by patients (B), reporting the classification and severity. Data are reported as the fraction of patients experiencing the AEs after treatment, classified in light, moderate, or extreme AEs.

Posttreatment adverse events (AEs) assessed by physicians were the following: acneiform eruptions, nodules (visible and/or palpable material), asymmetry, over‐ or under‐correction, needle mark, inflammatory nodule, Tyndall effect (blue bump), allergic reactions, granulomas, pain, erythema‐edema, ecchymosis‐hematoma, bruising, vascular occlusion, and Covid‐19 vaccine‐related hypersensitivity reaction. None of the treatments reported serious AEs (Figure 1A). The most commonly reported AE was pain, experienced by 8% of patients as light and by 4% in a moderate manner. Erythema and edema were the second most frequently occurring AEs, with 6% of patients experiencing them as light and 4% as moderate. Ecchymosis, bruising, and allergic reactions were each observed in 2% of the patients in a mild form. The allergic reactions, which were immediate, presented as urticaria‐like papules, redness, and itching. These symptoms were effectively managed with a short course of systemic steroids and antihistamines, administered over 3 days. It remains unclear whether the cause of the reactions was the injectable product itself or the adhesive taping (Tensoplast) used during the procedure. In one patient, a moderate infection was observed that led to pain and edema, which was resolved with antibiotics and steroids.

Patient‐reported AEs were mostly light in intensity (Figure 1B). The most experienced AE was pain during treatment, reported by 47% of patients in a light way, by 16% in a moderate, and in 2% (one patient) as extreme. After the treatment, soreness was mostly experienced AE (38%), however as mild and mostly resolving in the first 2 days after treatment (Table 3). Swelling was the second most experienced AE, with 69% of patients not reporting experiencing it. Other AEs reported as mild were bruising (4%), inflammation (2%), tenderness (8%), pain (8%), numbness (2%), and pulsing sensation (2%). Moderate AEs were reported for swelling, pulsing sensation, burning, daze, headache, and fever feeling, each by 4% of patients. Some patients did not report the intensity of the experienced AE or the duration, limiting the information available. However, no extreme AEs were reported by any patient. Most of the AEs resolved in the first 2 days after treatment (Table 3), with only some symptoms occurring in 3–4 days (swelling, soreness, bruising, tenderness, pain, and burning sensation). Infection reported by one patient resolved in the first week after treatment, as did tenderness, pain, numbness, and pulsing sensation, each reported by one patient. Only tenderness was observed for more than 8 days by one patient, resolved by the 10^th^ day after treatment. No AEs lasted more than 10 days.

**TABLE 3:** Adverse events as reported by patients after treatment.

AE/% of patients	No AEs	1–2 days	3–4 days	5–7 days	8–10 days	> 10 days
Swelling	69% (34/49)	22% (11/49)	2% (1/49)	0% (0/49)	0% (0/49)	0% (0/49)
Soreness	55% (27/49)	41% (20/49)	2% (1/49)	0% (0/49)	0% (0/49)	0% (0/49)
Bruising	94% (46/49)	0% (0/49)	2% (1/49)	0% (0/49)	0% (0/49)	0% (0/49)
Inflammation	98% (48/49)	0% (0/49)	0% (0/49)	2% (1/49)	0% (0/49)	0% (0/49)
Infection	100% (49/49)	0% (0/49)	0% (0/49)	0% (0/49)	0% (0/49)	0% (0/49)
Tenderness	88% (43/49)	6% (3/49)	2% (1/49)	2% (1/49)	2% (1/49)	0% (0/49)
Pain	90% (44/49)	2% (1/49)	4% (2/49)	2% (1/49)	0% (0/49)	0% (0/49)
Numbness	96% (47/49)	2% (1/49)	0% (0/49)	2% (1/49)	0% (0/49)	0% (0/49)
Tingling	100% (49/49)	0% (0/49)	0% (0/49)	0% (0/49)	0% (0/49)	0% (0/49)
Pulsing sensation	86% (42/49)	12% (6/49)	0% (0/49)	2% (1/49)	0% (0/49)	0% (0/49)
Burning	88% (43/49)	10% (5/49)	2% (1/49)	0% (0/49)	0% (0/49)	0% (0/49)
Daze	88% (43/49)	12% (6/49)	0% (0/49)	0% (0/49)	0% (0/49)	0% (0/49)
Headache	88% (43/49)	12% (6/49)	0% (0/49)	0% (0/49)	0% (0/49)	0% (0/49)
Fever feeling	88% (43/49)	12% (6/49)	0% (0/49)	0% (0/49)	0% (0/49)	0% (0/49)

**FIGURE 2:**
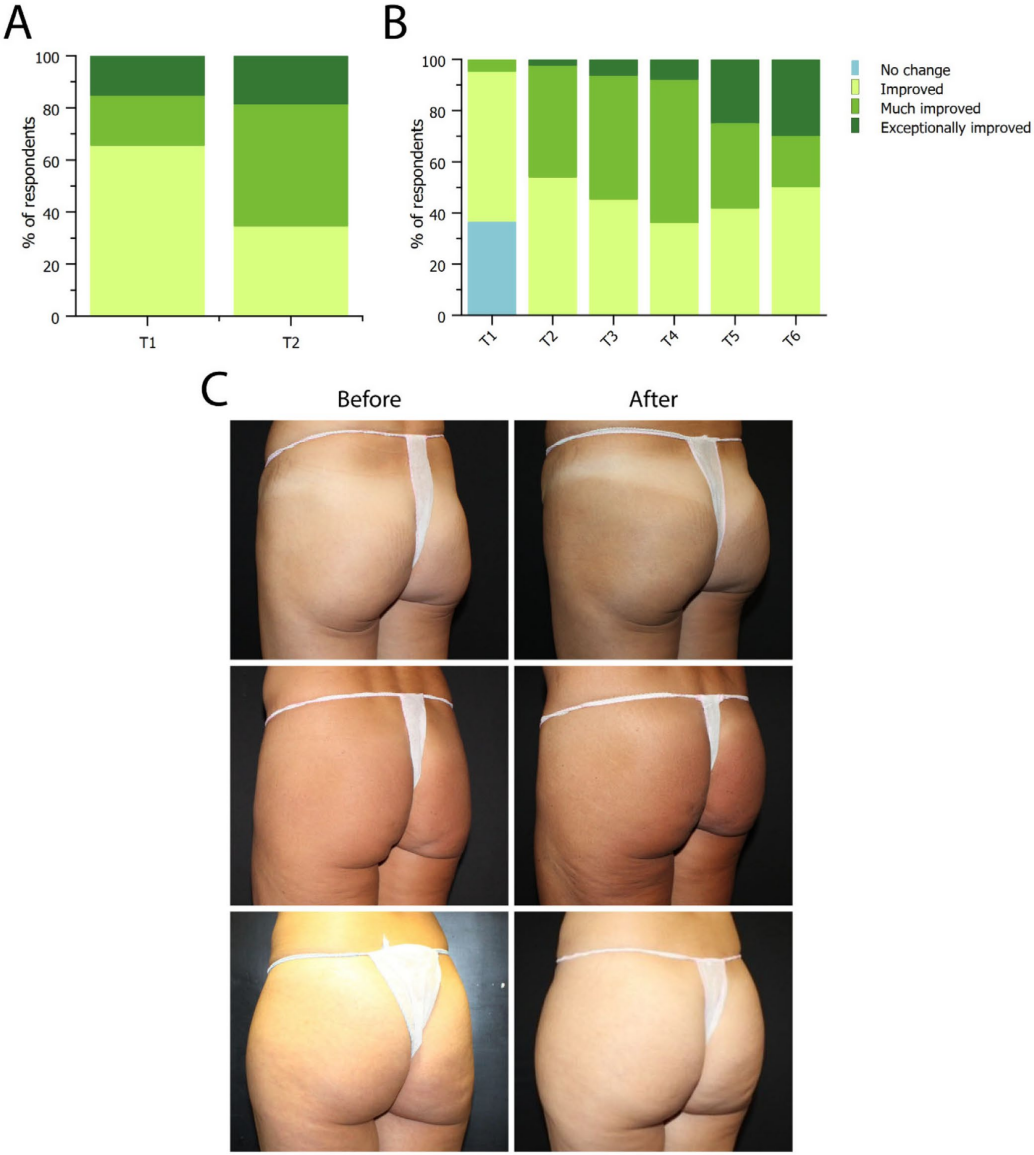
Global Aesthetic Improvement Scale (GAIS) assessment by patients (A) and physicians (B) immediately after treatment (T1) and at follow‐ups. Data are reported as the fraction of physicians and patients reporting the perceived level of improvement after treatment on a scale ranging from “no change” to “exceptionally improved.” (C) Before and after treatment with GANA X, performed twice with a treatment interval of 6–8 weeks.

As reported through the Global Aesthetic Improvement Scale (GAIS), both physicians and patients observed an improvement after the treatment (Figure 2). In the case of patients, a significant improvement was reported already immediately after treatment (T1, Figure 2A), that ameliorated further at the follow‐up visit (T2). Physicians also reported a substantial improvement that increased over time, as observed in the following follow‐up visits up to 24 months after the first treatment (Figure 2B). In summary, no treatment resulted in a lack of change, and the trend indicates that results are ameliorating over time. Treatment results are provided in representative pictures of before and after treatment in Figure 2C.

**FIGURE 3:**
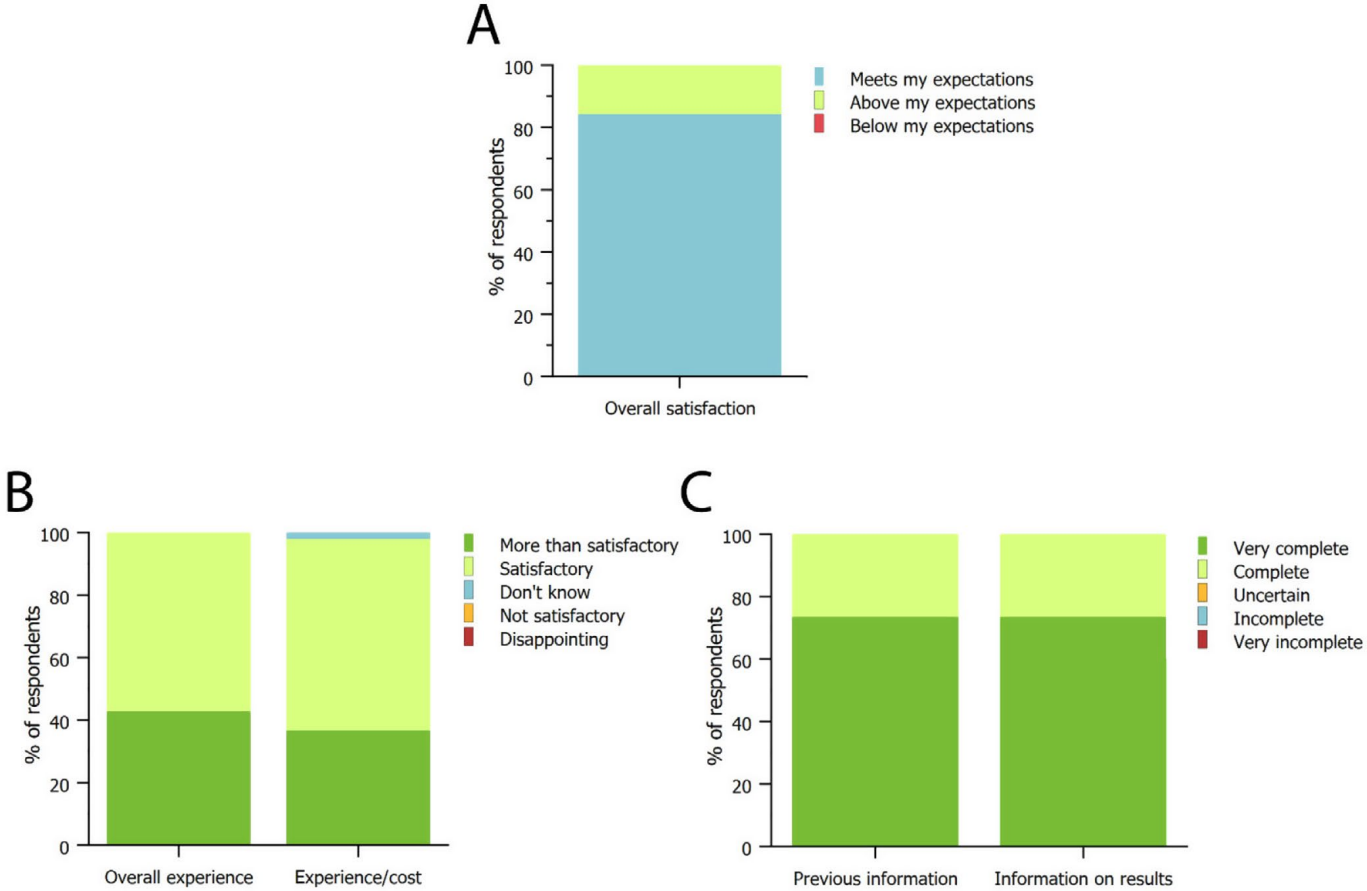
Treatment satisfaction was evaluated by surgeons (A) and patients (B), who were also asked how they valued the results based on treatment costs. (C) Level of completeness of information provided to the patients regarding the treatment modalities and the expected results before the treatment. Data are reported as the fraction of physicians and patients answering the survey.

Based on physicians' experience, results were all meeting the treatment expectations, with 16% reporting that it was above their expectations (Figure 3A). All patients reported to be satisfied by the overall experience, with a 43% being more than satisfied. Even when considering the economic side, almost all patients were satisfied by the results relatively to the cost, with 37% being more than satisfied (Figure 3B). All patients reported they were well informed about the procedure and results, with the majority reporting they received very complete information (Figure 3C).

**FIGURE 4:**
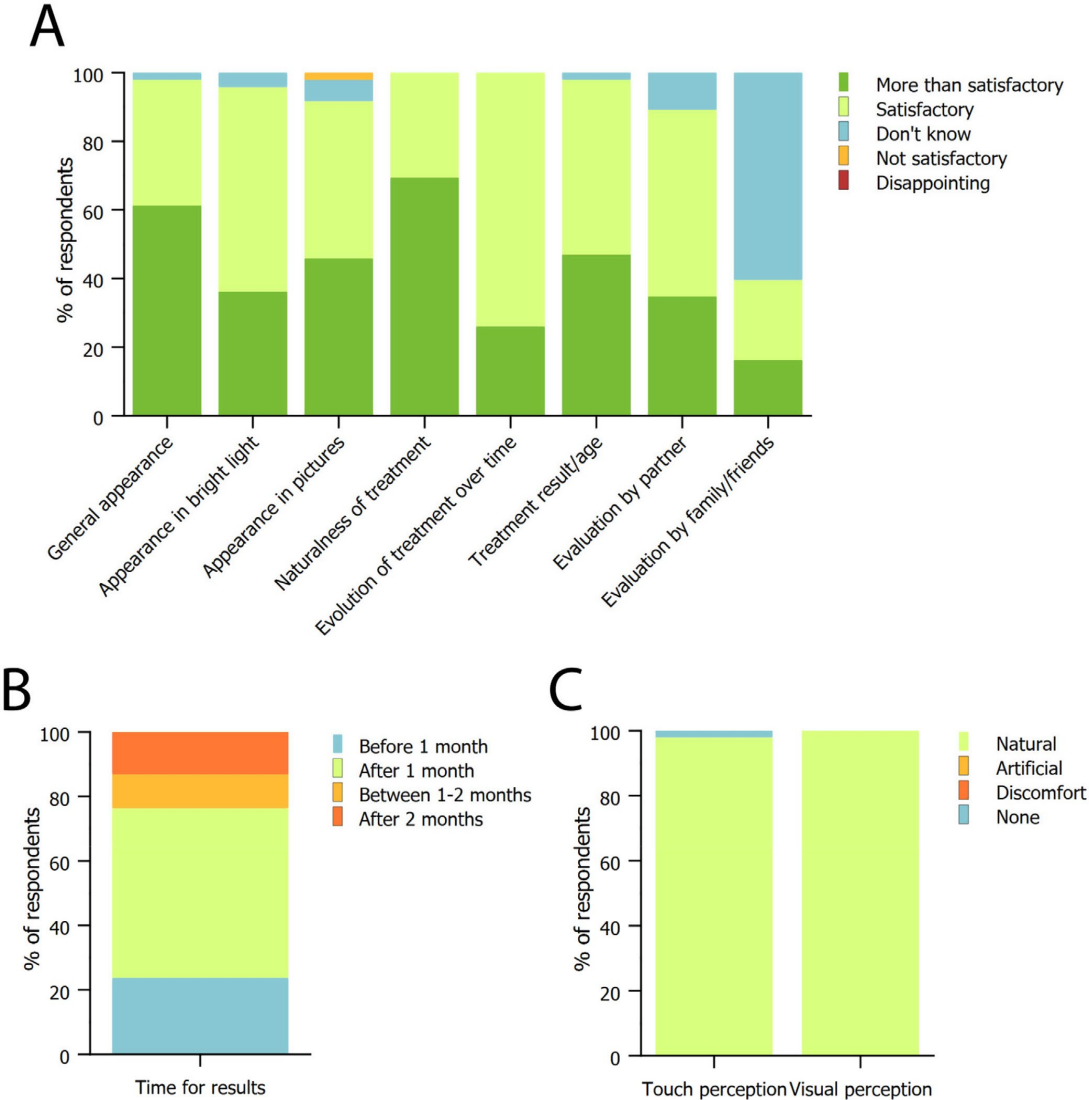
Patients in‐detail evaluation on treatment results. (A) Evaluation of the external appearance of the treated area, considering the general appearance, naturalness, evolution, result compared to patients' age, and external evaluation by partners, family, and friends. (B) Patient‐reported timeframe for results to be visible after treatment. (C) Patient‐reported perception of results at touch or at sight. Data are reported as the fraction of patients answering the survey.

Patients were also asked how they rated the overall appearance of the gluteal area. Most of the patients were satisfied with the general appearance of the treated area, as well as how it appeared in bright light and in pictures. All patients agreed that the treatment looked natural and that it improved over time. Improvement rates were similar between younger and older patients, with the latter needing a couple more sessions to reach the desired result. However, results were comparable after 1 year, and just needed a single yearly session to maintain the results. Patients' partners also agreed that the result was satisfactory, whereas the majority of patients did not interrogate family or friends about the result, although the ones who did it received positive feedback (Figure 4A). Regarding the time needed to appreciate the result, most of the patients reported that it took more than 1 month (Figure 4B), but that the delay in seeing visible improvement was a problem only for a small fraction of patients (10%). The appearance of the treatment was natural for almost all patients both at touch and at visual perception (Figure 4C).

**FIGURE 5:**
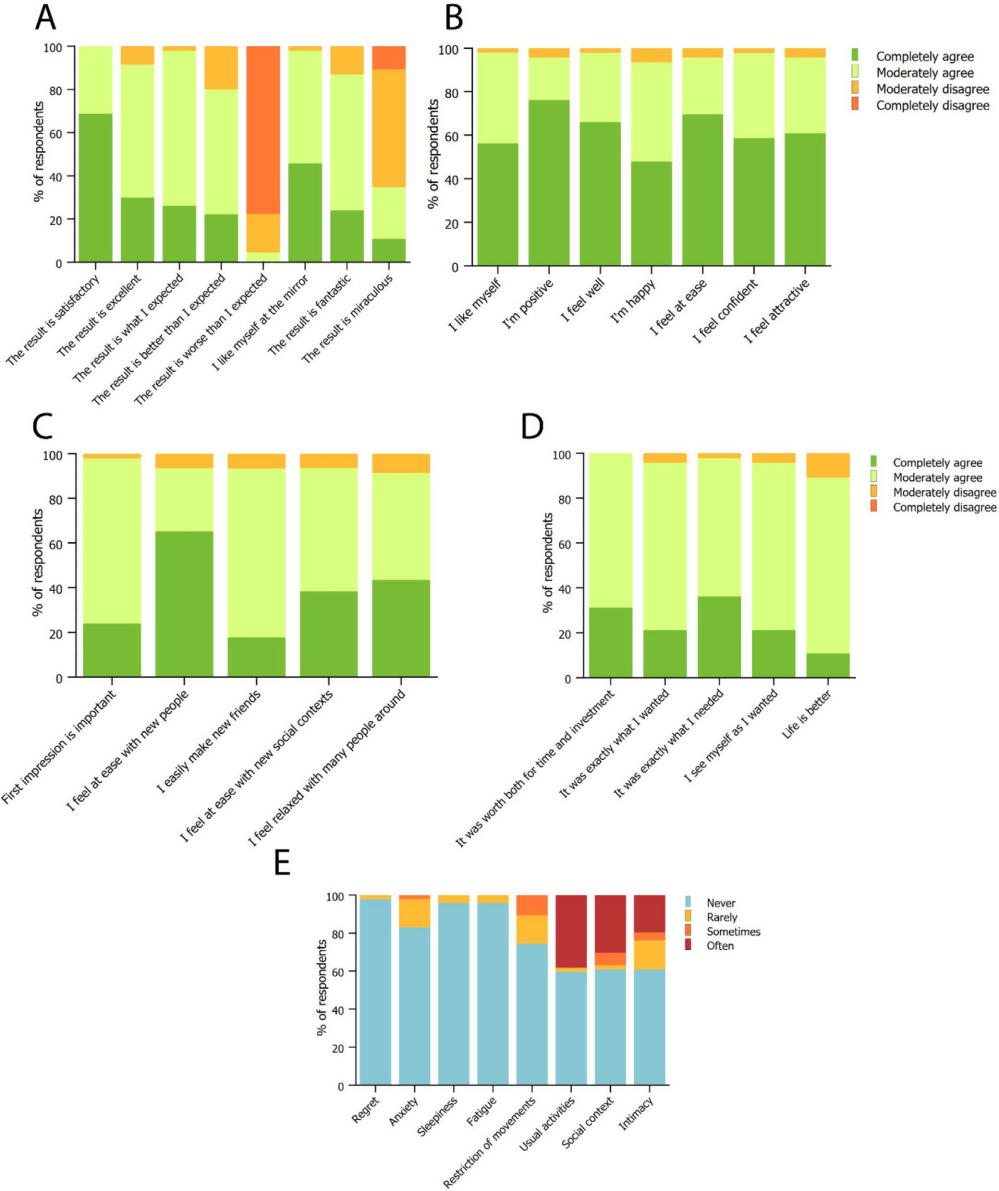
Patients' evaluation on quality of life after treatment. (A) Satisfaction regarding results and expectations; (B) evaluation of treatment's impact on self‐esteem; (C) evaluation of treatment's impact on social life; (D) evaluation of treatment's worth and importance on patients' lives; (E) effect of treatment in the first days regarding feelings of regret, anxiety, sleepiness, fatigue, restriction of movements, usual activities, social context, and intimacy. Data are reported as the fraction of patients answering the survey.

Patients were then asked their agreement level on treatment results and its effects on their lives. All patients agreed that the result is satisfactory, and for most of the patients even beyond their expectations. Almost all the patients reported that they like themselves at the mirror regarding treatment result (Figure 5A). When asked about self‐esteem, most of the patients answered that they like themselves, are positive, feel well, are happy, feel at ease, feel confident, and feel attractive, indicating an overall high self‐confidence after the treatment (Figure 5B). Regarding social life behavior, almost all patients agreed on the importance of the first impression. The majority of patients reported that they feel at ease with new people and they easily make new friends, they feel at ease in new social contexts and they feel relaxed with many people around (Figure 5C). The sentiment on the decision to perform the treatment was for all patients worth both the time and investment. Almost all patients agreed that the treatment was exactly what they wanted and needed, and they now see themselves as they wanted. The majority agreed that their life is now better (Figure 5D). The impact of the treatment on the first days afterward almost never led to feelings of regret, sleepiness, or fatigue, but it rarely caused some anxiety and restriction of movements. The treatment had an impact in a third of the patients on usual activities, social context, and intimacy (Figure 5E).

Most of the patients stated that the treatment impacted on their self‐esteem, improving it. Just 22% of patients reported that the treatment influenced the relationship with the partner, and 7% with friends and family, all in a positive manner (Table 4). The majority of patients would repeat the treatment at 1 year (only one would not, only because the treatment was already evaluated as extremely effective) and all of them would recommend it to friends.

**TABLE 4:** Self‐esteem and impact on relationship survey following treatment.

	(%) (*n* pat/tot *n* pat)
Did the treatment affect your self‐esteem?	
Yes	79% (37/47)
No	22% (10/47)
If yes, how?	
It increased a lot	13% (6/47)
It increased	64% (30/47)
It decreased	0% (0/47)
It decreased a lot	0% (0/47)
I don't know	2% (1/47)
Did the treatment affect your relationship with the partner?	
Yes	22% (10/46)
No	78% (36/46)
Did the treatment affect your relationship with family and friends?	
Yes	7% (3/43)
No	94% (40/43)

## 4 Conclusions

Poly‐l‐lactic acid (PLLA) biostimulators are getting increasing attention in the field of aesthetic medicine due to their biocompatibility and long‐lasting effects. Although their principal application is for facial rejuvenation, their off‐label applications in other parts of the body are being studied, with brilliant results both regarding efficacy and safety. The ease of use and comparatively lower costs makes this class of fillers a valuable alternative to surgery or lipofilling in volume augmentation, contouring, and other types of body rejuvenation procedures. Unlike conventional fillers, PLLA achieves its effects through the stimulation of the patient's own collagen. The biostimulator works through the activation of a subclinical inflammatory response, followed by encapsulation and fibroplasia that results in the desired aesthetic effect [11]. PLLA stimulators achieve a remarkable effect even in aged skins, where they elicit collagen deposition through M2 polarization of resident macrophages, that in, turn activate fibroblast‐mediated collagen deposition [26]. The collagen deposition process is timely, requiring 8–24 months to be achieved, depending on the patient's age and oxidative status. Thus, PLLA treatments are usually administered several weeks or months apart, to ensure an even deposition and avoid overcorrection [27].

In this study, we showed that the use of GANA X, a PLLA‐based filler, is safe and effective in buttocks treatment for volume augmentation, cellulite dimples, contour irregularities, and skin tone and texture improvement. The results were reported independently by both patients and physicians in a survey, evaluating both treatment efficacy in improving aesthetic appearance and evaluating AEs.

Most patients did not experience any AE, with the majority being mild and resolving spontaneously after a few days. Some pain was experienced during treatment, but only in one case, it was described as extreme. Soreness was the most common AE after treatment, occurring only mildly and resolving completely 2 days after treatment. Other experienced AEs were swelling, bruising, inflammation, infection, tenderness, pain, numbness, pulsing sensation, burning, daze, headache, and fever feeling. However, each of these AEs was experienced only by a small fraction of patients, and mostly in a mild intensity. No patient experienced extreme AEs, and no AE lasted more than 10 days, proving the safety of this method. Patients were prescribed with mild NSAIDs within the following 24 h according to physician's experience, to ameliorate compliance to the treatment. The good outcomes of the procedure were ensured by physicians following the GANA X proposed precautions. These include cleaning the injection area with an antiseptic disinfectant, closing the entry points with sterile plaster, and taping the area for 24 h. An elastic dressing was suggested also for the first 24 h.

The aesthetic improvement after treatment was confirmed by both physicians and patients, ranging from improved to exceptionally improved, with long‐lasting effects recorded at subsequent follow‐up visits up to 24 months. The trend of exceptionally improved treatments increased over time, indicating an effect of multiple injections as well as the progressive action of remodeling that relies on patients' own collagen deposition. Most of the patients declared that results started to be visible after 1 month of treatment but were mild, in line with the proposed mechanism of action [28]. The result was evaluated as completely satisfactory at 1 year post‐follow‐up and was declared as natural both at sight and at touch. The impact on patients' life was overall very positive, as confirmed with increased self‐esteem and quality of life after treatment. Overall, all patients were satisfied by the results and the whole experience, even when considering the costs. A possible treatment repetition after 1 year was suggested by physicians and considered by many patients to consolidate the treatment results, that can then reach 2–3 years. A shorter timeframe to repeat the treatment is not suggested due to the PLLA biostimulation process, which takes place for 8–9 months, and could thus result as an overcorrection.

A potential limitation of this study is the limited number of patients enrolled and treated. However, the high level of satisfaction both by physicians and patients suggests that GANA X has excellent efficacy and tolerance profiles for buttocks treatment.

In conclusion, the use of PLLA filler GANA X for buttocks is encouraged due to the provided results and should be evaluated for other areas as thighs, arms, and other body laxities, offering an exceptional alternative to standard aesthetic surgery.

## Author Contributions

P.R.R., B.B., F.D.A., R.F., F.V., and G.S. designed and performed the research. P.R.R., B.B., F.D.A., R.F., F.V., and G.S. analyzed the data and wrote the paper. All authors have read and approved the final manuscript.

## Ethics Statement

The authors have nothing to report.

## Consent

Proper written informed consent was obtained from each patient.

## Conflicts of Interest

The authors declare no conflicts of interest.

## Data Availability

The data that support the findings of this study are available from the corresponding author upon reasonable request.
